# Simultaneous Epicardial and Noncontact Endocardial Mapping of the Canine Right Atrium: Simulation and Experiment

**DOI:** 10.1371/journal.pone.0091165

**Published:** 2014-03-05

**Authors:** Sepideh Sabouri, Elhacene Matene, Alain Vinet, Louis-Philippe Richer, René Cardinal, J. Andrew Armour, Pierre Pagé, Teresa Kus, Vincent Jacquemet

**Affiliations:** 1 Département de Physiologie, Université de Montréal, Montréal, Québec, Canada; 2 Centre de Recherche, Hôpital du Sacré-Coeur de Montréal, Montréal, Québec, Canada; 3 Conseil National de Recherche Canada, Montréal, Québec, Canada; 4 Département de Pharmacologie, Université de Montréal, Montréal, Québec, Canada; 5 Department of Pharmacology, East Tennessee State University, Johnson City, Tennessee, United States of America; 6 Département de Chirurgie, Université de Montréal, Montréal, Québec, Canada; University Hospital of Würzburg, Germany

## Abstract

Epicardial high-density electrical mapping is a well-established experimental instrument to monitor *in vivo* the activity of the atria in response to modulations of the autonomic nervous system in sinus rhythm. In regions that are not accessible by epicardial mapping, noncontact endocardial mapping performed through a balloon catheter may provide a more comprehensive description of atrial activity. We developed a computer model of the canine right atrium to compare epicardial and noncontact endocardial mapping. The model was derived from an experiment in which electroanatomical reconstruction, epicardial mapping (103 electrodes), noncontact endocardial mapping (2048 virtual electrodes computed from a 64-channel balloon catheter), and direct-contact endocardial catheter recordings were simultaneously performed in a dog. The recording system was simulated in the computer model. For simulations and experiments (after atrio-ventricular node suppression), activation maps were computed during sinus rhythm. Repolarization was assessed by measuring the area under the atrial T wave (ATa), a marker of repolarization gradients. Results showed an epicardial-endocardial correlation coefficients of 0.80 and 0.63 (two dog experiments) and 0.96 (simulation) between activation times, and a correlation coefficients of 0.57 and 0.46 (two dog experiments) and 0.92 (simulation) between ATa values. Despite distance (balloon-atrial wall) and dimension reduction (64 electrodes), some information about atrial repolarization remained present in noncontact signals.

## Introduction

Despite decades of investigations, many questions related to the mechanisms underlying the initiation and maintenance of atrial fibrillation remain open [1]. The origin of atrial fibrillation may be neurogenic (the role of the intrinsic cardiac nervous system is critical in this case), myogenic (related to abnormalities or remodeling in the heart muscle), or due to a combination of both [2]. Animal models have been developed to investigate the relation between atrial fibrillation and the autonomic nervous system [3]. In these models, atrial tachyarrhythmias could be induced by electrically stimulating the vagosympathetic trunks in the neck [4] or mediastinal nerves [5], [6]. Heterogeneity of repolarization was created locally in various atrial areas depending on which nerve was stimulated. Tachyarrhythmia beats typically originated from areas of increased repolarization heterogeneity. In animal models reproducing the neurogenic form of atrial fibrillation, the identification of neurogenically-induced repolarization gradients in sinus rhythm is critical for understanding the mechanisms of arrhythmogenicity.

The area under the atrial repolarization wave (ATa) in unipolar electrograms has been proposed as a marker of neurogenically-induced repolarization gradients [7]. This measure provides a simple way of assessing *in vivo* changes in atrial repolarization from electrical mapping signals and is more robust than the amplitude of the atrial T wave used to detect atrial repolarization alternans [8], [9]. It enables the monitoring of transient variations in repolarization in response to nerve stimulation preceding the onset of atrial fibrillation [10] without electrically stimulating the myocardium. The importance and spatial distribution of the influence of the autonomic nervous system on atrial repolarization has been demonstrated by measuring ATa in epicardial mapping data [10] and is supported by computer simulations [11], [12]. However, anatomical landmarks (fat pads, veins and arteries) caused obstruction to epicardial plaque placement, leaving relevant regions (e.g. septum) unmapped. To circumvent that problem, the use of noncontact endocardial mapping has been proposed [10]. Noncontact mapping [13], [14] is a clinical device that computes virtual endocardial electrograms from a multielectrode array (balloon catheter) inserted in the atrium. This tool has been applied to assess reentrant circuits [15]–[17] and focal activity [18], [19].

Activation and isopotential maps derived from virtual endocardial electrograms have been compared to direct-contact mapping in human [20]–[22] and canine atria [23]–[25]. Contact and non-contact electrogram morphology were compared in terms of correlation coefficients. In these studies, however, due to the presence of far-field ventricular activity, either atrial repolarization was ignored in the morphology comparison or the ventricular artifacts were included for computing the correlation. As a result, little is known about the potential of noncontact mapping for extracting information about atrial repolarization.

The aim of this paper is to evaluate the feasibility of the use of noncontact mapping for extracting activation times and ATa values in the context of dog experiments in a structurally normal heart. We hypothesized that some information about atrial repolarization remains present in noncontact signals. Dog experiments and a computer model of the canine right atrium extending the work of Voth [26] were combined to test this hypothesis. Simultaneous epicardial and noncontact endocardial mapping were compared in their ability to describe atrial depolarization and repolarization. The computer model was used to estimate the loss of information resulting from the inverse problem alone (contact vs noncontact).

## Materials and Methods

### Ethics Statement

All experiments were performed in accordance with the guidelines for animal experimentation and were approved by the ethical committee of the Hôpital du Sacré-Coeur de Montréal.

### Animal Preparation

Two large mongrel dogs (about 35 kg) were included in this pilot study. The first dog was used to create the computer model and the second to give some sense of experimental reproducibility. Each dog was anesthetized by sodium thiopental and maintained under positive pressure ventilation. After bilateral open chest surgery to expose the heart, anesthetic was changed to α-chloralose. Atrioventricular blockade was performed by formaldehyde injection to dissociate atrial and ventricular electrical activity, as in Armour *et al*. [10]. The right ventricle was electrically stimulated at 82 bpm to assure sufficient cardiac output. The atria were in sinus rhythm during the whole experiment.

### Experimental Recording System

Two silicone plaques comprising 103 epicardial unipolar recording contacts were placed (1) in the right atrial free wall and lateral right atrial appendage (79 channels), and (2) in the Bachmann’s bundle and adjacent base of the medial atrial appendage (24 channels) [10]. The electrodes were connected to a multi-channel recording system (EDI 12/256, Institut de génie biomédical, École Polytechnique de Montréal). Signals were band-pass filtered (0.05–450 Hz) and digitized with a sampling rate of 1 kHz.

**Figure 1:**
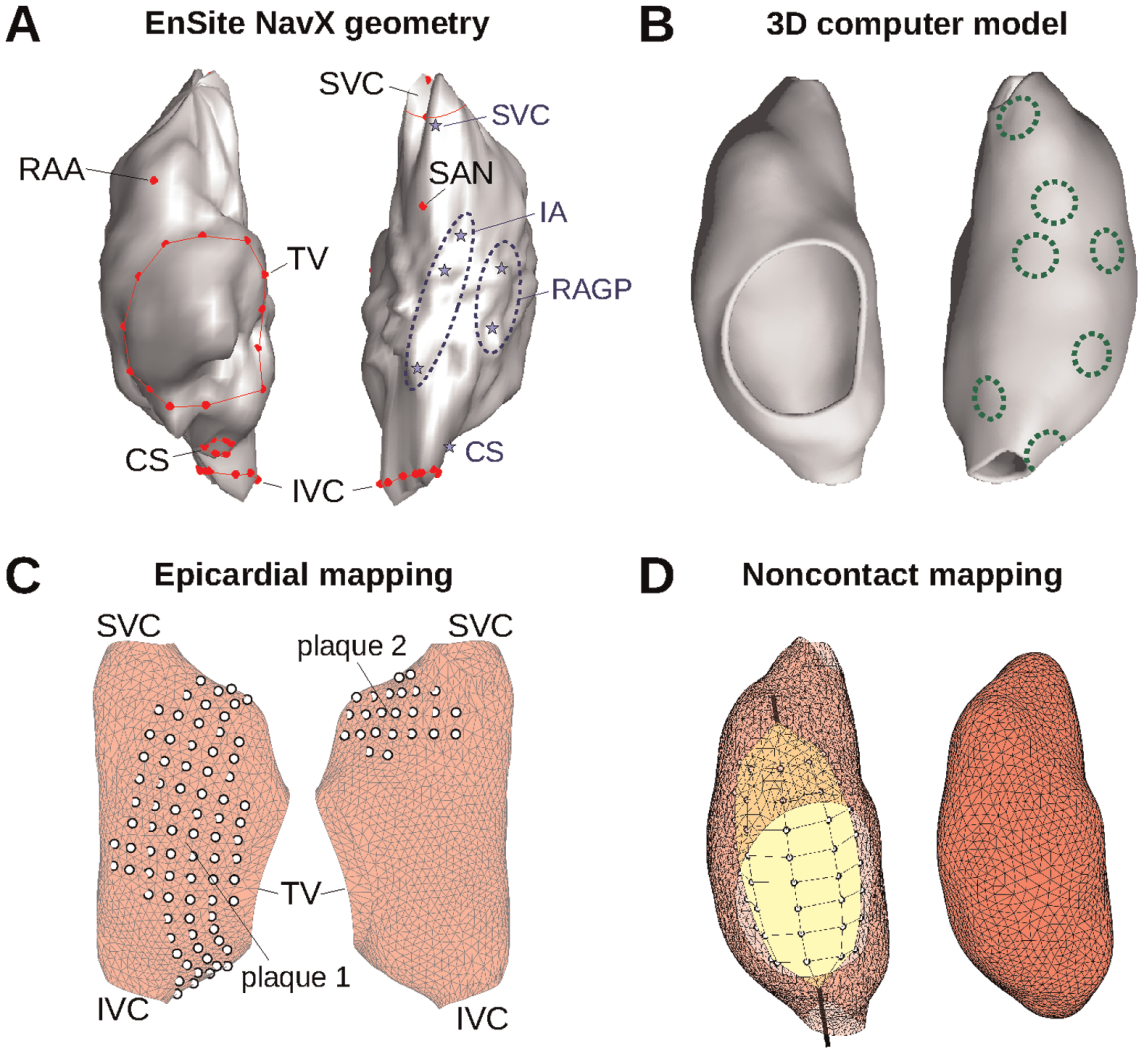
Right atrium geometry and electrode configuration. (A) Endocardial surface of a canine right atrium as reconstructed by the EnSite NavX system (*left side*: anterior view; *right side*: posterior view). Anatomical features identified by the catheter localization system are shown in red. Blue stars represent recording sites of the direct-contact endocardial catheter (B) 3D geometrical model (same views as panel A) of the right atrium after processing. Dashed circles represent the location of heterogeneity regions, shown here with a radius of 3 mm. (C) Epicardial electrode position for the two plaques in the computer model. (D) *Left side*: Balloon catheter with its 64 electrode. *Right side*: closed endocardial surface used for the inverse problem. RAA: right atrium appendage; SVC: superior vena cava; IVC: inferior vena cava; TV: tricuspid valve; CS: coronary sinus; SAN: sino-atrial node; RAGP: right atrium ganglionated plexus; IA: inter-atrial bundles.

In parallel, a noncontact, endocardial balloon catheter (EnSite 3000 Multi Electrode Array with 64 channels; St Jude Medical Inc., St Paul, MN) was inserted in the right atrium. This device solves an inverse problem (see below) to compute endocardial electrograms at 2048 sites on a virtual closed surface representing the endocardium based on the potential at the 64 electrodes of the balloon [27]. This procedure requires reconstructing the endocardial geometry using a catheter localization system (EnSite NavX electroanatomical navigation system). Reconstruction of atrial geometry is shown in Fig. 1A for the dog considered in this study. The acquisition system outputs 2048 (virtual) endocardial electrograms as well as 3-lead ECG at a sampling rate of 1.2 kHz.

A second catheter in the right atrium served to measure direct-contact endocardial bipolar electrograms. This catheter, also localized and tracked by the system, was moved to record signals at 7 different locations (>5 sec stable recording at each location) near the superior vena cava, the right atrium ganglionated plexus, the inter-atrial bundles and the coronary sinus (stars in Fig. 1A).

Epi- and endocardial signals were simultaneously recorded using a separate digital acquisition system. To enable their synchronization, both systems had a “clock” input channel connected to a manually-driven tick generator.

### Simulation of Electrical Propagation in the Right Atrium

The geometry extracted by the Ensite NavX system (Fig. 1A) formed the basis for constructing a 3D model of the canine right atrium. The dog that had the most accurate geometrical reconstruction (based on more acquisition points) was used for that purpose. The triangulated surface was processed and smoothed using VRMesh (VirtualGrid, Bellevue City, WA). Holes corresponding to the superior and inferior vena cava and to the tricuspid valve were created based on several points around their circumference identified using the catheter localization system (Fig. 1A). A thin-walled 3D cubic mesh (wall thickness: 1.75 mm; spatial resolution 0.25 mm; see Fig. 1B) was generated from the resulting triangulated surface as in our previous works [28]. Fiber orientation was specified following a rule-based approach [29]. There was no attempt to reproduce fine anatomical details and the trabecular structure of the right atrium (terminal crest and pectinate muscles) since no preparation-specific information was available for these anatomical features. Due to the limited spatial resolution of the NavX system, the details of the right atrium appendage anatomy were only grossly incorporated, as in older models [30].

These model limitations were deliberately introduced in order to ensure that: (1) there will be no real difference in epicardial and endocardial activation in the model; (2) the appendage (not taken into account by the Ensite device) will have a small influence on the simulation results. Consequently, the simulations represent a best-case scenario in which the loss of information due to the noncontact electrodes will be isolated from the effects of trabecular structure, epicardium-endocardium dissociation and heart contraction. These additional features may further reduce the accuracy of noncontact mapping.

Electrical propagation was simulated by solving the monodomain equation [31] in the cubic mesh using finite difference methods [32]. In the context of normal propagation in the absence of structural heart disease (e.g. fibrosis), this formulation, also used in previous works on atrial repolarization wave simulation [12], was shown to be sufficiently accurate, including for the computation of electrical signals [33]. Explicit time integration with a time step of 20 µs was used. Membrane kinetics was described by the Ramirez *et al*. model of canine atrial cell [34], [35]. Effective tissue conductivity was 12 mS/cm (longitudinal), anisotropy ratio was 3∶1, and membrane surface-to-volume ratio was 2000 cm^−1^. Sinus rhythm propagation was elicited by injecting intracellular current at the anatomical location corresponding to the sino-atrial node (or more precisely to the focal point of activation) as identified using the catheter localization system (Fig. 1A).

To introduce repolarization heterogeneity in a way that replicates the Kneller *et al*. model of cholinergic atrial arrhythmia [34], we created circular zones of heterogeneity in membrane properties (Fig. 1B). Similarly to Vigmond *et al*. [11], zone radius was varied between 2 and 5 mm. In these zones (one at a time), acetylcholine (ACh) concentration was set to 0.03 µM based on Kneller *et al*. [34]. The resulting increase in ACh-dependent K+ current significantly shortened action potential durations in the zone, thus creating repolarization gradients [11].

For each substrate (control +7 zone locations × 3 zone radius = 22 simulations), sinus rhythm with a stable cycle length of 600 ms was simulated (experimentally-measured cycle length was 595±9 ms at baseline). Simulations were run until steady-state was reached, as determined by convergence of action potential durations (beat-to-beat variation <1%). Analysis was performed on the last simulated beat.

### Simulation of Epicardial Electrograms

Epicardial mapping (“plaques”) was simulated using the same tools as in Jacquemet *et al*. [36]. Two plaques were used, as in the experiment: one in the right atrium free wall and one between Bachmann’s bundle and the appendage. Electrode configuration reproducing each experimental plaque was projected on the atrial epicardial surface on the basis of three manually-positioned control points (two electrodes at the extremities and one at the center of the plaque). The configuration is shown in Fig. 1C.

Electric potential at each of the 103 unipolar electrodes was computed using the current source approximation as in previous works [12], [36]. In this framework assuming an infinite uniform volume conductor, the potential *φ* of an electrode located at **x** is given by
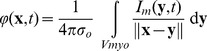
(1)where is σ*_o_* the extracellular conductivity, *I_m_* is the transmembrane current computed from the time course of the membrane potential in all simulated cardiac cells, and *Vmyo* is the integration domain (myocardium) [31]. The same formula (1) was used to compute potentials in the endocardium and in the blood cavity (see below).

### Simulation of Noncontact Endocardial Electrograms

Since the EnSite software is proprietary, the noncontact mapping system was simulated using not exactly the same method, but a conceptually similar one based on Harley *et al.* [37]. In these approaches, the atrial geometry is specified by a closed surface *S* near the endocardium and whose interior *V* contains only blood. The surface *S* used by the EnSite software for the experiments is displayed in Fig. 1A; the one used for the simulations is shown on the right side of Fig. 1D. The 64 electrodes of the Ensite Array Catheter are located at ***x*** *_i_*, *i* = 1 to 64, all inside the surface *S* (Fig. 1D, left side). Potentials at these 64 electrodes were computed using Eq. (1). Assuming homogeneity and isotropy of blood conductive properties and neglecting the effect of the catheter on volume conduction, the electric potential *φ* satisfies the Laplace equation Δ*φ*  = 0 in *V*.

Because of the uniqueness of the solution to the Laplace equation with Dirichlet boundary conditions, the values of *φ* on *S* determine the value of *φ* at x*_i_*, denoted by 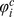
(*c* stands for catheter). An explicit formula can be derived from potential theory. From the Green’s second identity, if x is in the interior of *V*, then [37]

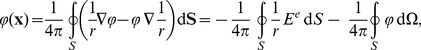
(2)where *r* is the distance between **x** and the surface element d*S*, *E^e^* is the normal component of 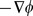
with respect to the surface *S* (the index *e* stands for endocardium), and 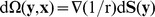 is the solid angle subtended at **x** by the element d**S** located at **y** [31].

In order to compute these integrals numerically, *S* is discretized as a triangulated surface with *N* nodes located at **y** *_j_*, *j* = 1 to *N*, where *N* is of the order of 2000. The field *φ* is approximated on the endocardium surface *S* using piecewise linear basis functions *ψ_j_*:
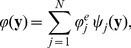
(3)where 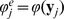. If the 64-by-*N* matrices **O** ^ec^ and **S** ^ec^ are defined as:
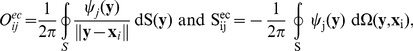
(4)then (2) can be written as



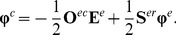
(5)Similarly, if **x** is on the boundary *S*, because of the singularity at *r* = 0,
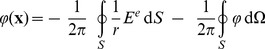
(6)and by defining the *N*-by-*N* matrices **O** ^ee^ and **S** ^ee^ as
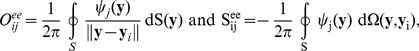
(7)the potential on the surface satisfies the equation



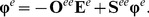
(8)After E*^e^* is isolated in (8) and substituted in (5), the potential at the catheter electrodes is expressed as 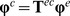, where the 64-by-*N* forward transfer matrix is given by
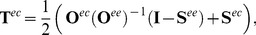
(9)where **I** is the identity matrix. Since the function *ψ_j_* is linear on every triangle, the integrals from (4) and (7) are finite and can be computed analytically [38], [39], including in the presence of a singularity (*i.e*. when r = 0 in the integration domain). The auto-solid angle 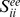 is defined such that the sum of each row of **S** *^ee^* gives 1.

To estimate the potential at the endocardium from the potential at the catheter electrodes, the forward transfer matrix needs to be inverted. Because the system is underdetermined, Tikhonov regularization [31] was used to compute the inverse transfer matrix **T** *^ce^*:
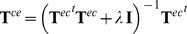
(10)where λ is a positive regularization parameter. The parameter λ was set to 1.4 ·10^−5^ based on a comparison between electrograms computed directly using Eq. (1) and those obtained by solving the inverse problem.

### Processing of Atrial Electrograms

Atrial activation times were identified in both epi- and endocardial electrograms using a dedicated event detector based on signal derivative [40]. Activation maps were validated manually by visual inspection of electrogram waveforms and activation times. Noncontact mapping sometimes produced fractionated endocardial waveforms (double potentials) that reduced the accuracy of detected activation times. To cope with that limitation, spatial filtering (Gaussian filter with a space constant of 5 mm) was applied to the resulting endocardial activation maps.

For each ventricular activation in the experimental signals, the onset of the Q wave and the offset of the T wave were identified manually on the ECG. For each atrial activation, the atrial activity interval was defined as the time interval between the earliest atrial activation time and the latest atrial activation +300 ms, assuming that atrial action potential durations were always shorter than 300 ms (which was *a posteriori* verified by inspecting the atrial T waves). Only atrial beats for which the atrial activity interval did not overlap with any QT interval were considered for subsequent analysis, in order to prevent the contamination of atrial signals by ventricular activity. This issue was not present in the simulated signals.

To quantify the local repolarization gradient, the area under the atrial T wave [7], [12] (ATa) was computed. The (non-dimensional) ATa of an electrogram waveform *φ* (*t*) was defined as:
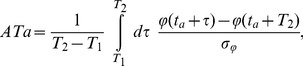
(11)where *t_a_* is the activation time and σ*_φ_* is the standard deviation of the whole signal *φ*. The integration bounds *T*_1_ and *T*_2_ were initialized to 50 and 300 ms. Further manual validation was performed to ensure appropriate positioning of integration bounds. After minor manual adjustments (mostly for the lower bound), the interval length *T*_2_–*T*_1_ was respectively 256±20 ms and 254±16 ms in epi- and endocardial experimental signals. For simulated signals, the bounds were set to *T*_1_ = 35 ms and *T*_2_ = 300 ms. Normalization by interval length was aimed at providing a non-dimensional ATa. It did not significantly influence the results since interval length was essentially the same across all electrodes. Amplitude normalization compensated epi-endo and between-channels amplitude differences that may be generated by the inverse problem solver, and facilitated the comparison between simulations and experiments. Baseline correction applied in (11) assumed that the point at the upper integration bound was isoelectric (just after the end of atrial repolarization and before the next onset of the Q wave). Since the previous and next atrial beats were typically partially masked by ventricular activity no interpolation of the isoelectric line was possible.

### Correspondence between Epicardial and Endocardial Maps

One issue was to determine which endocardial electrode was closest to each epicardial electrode. In the computer model, this task was simply performed by identifying the endocardial electrode that minimized the Euclidian distance to the given epicardial electrode. In the experiment, the locations of epicardial electrodes in the EnSite endocardial coordinate system were not accurately known. The approximate location of some of the electrodes was however obtained using the catheter localization system.

The epi-endo correspondence was reconstructed iteratively. The grid of electrodes (plaques) was created based on three control points (like for the simulation of epicardial mapping, see above). The location of these three points was initialized using *a priori* knowledge about plaque placement. The position of these three points was then adjusted by random search within circular regions of diameter 5 mm around each initial control point position. The remaining points were positioned on the basis of the grid configuration, assuming regular inter-electrode spacing (this is similar to rigid registration). The optimization criterion was the root mean squared (RMS) difference between activation times in the epicardial map and at the corresponding electrodes of the endocardial map, summed up over three carefully-validated beats. Since anatomical reconstruction of the appendage was not accurate, electrodes located in the appendage were excluded in the computation of the optimization criterion.

Epicardial and endocardial maps were quantitatively compared using Pearson’s (product-moment) correlation coefficients [41] computed using Matlab. The software also outputted a p-value to exclude the hypothesis of no correlation.

## Results

### Activation Maps

During the experiment in the first dog, the RR interval was 733±2 ms (ventricular pacing) and the QT interval was 353±22 ms. As a result, there were sequences of 380-ms intervals free of ventricular activity, while atrial depolarization time was <50 ms in the mapped area and repolarization always lasted less than 250–300 ms. In total, 12 atrial beats were found in these intervals free of ventricular activity. At least one suitable beat was identified for each of the 7 recording sites of the endocardial catheter. In the second dog, the RR interval was 733±6 ms (pacing) and the QT interval was 398±16 ms. Nine suitable beats were selected for the analysis.

**Figure 2:**
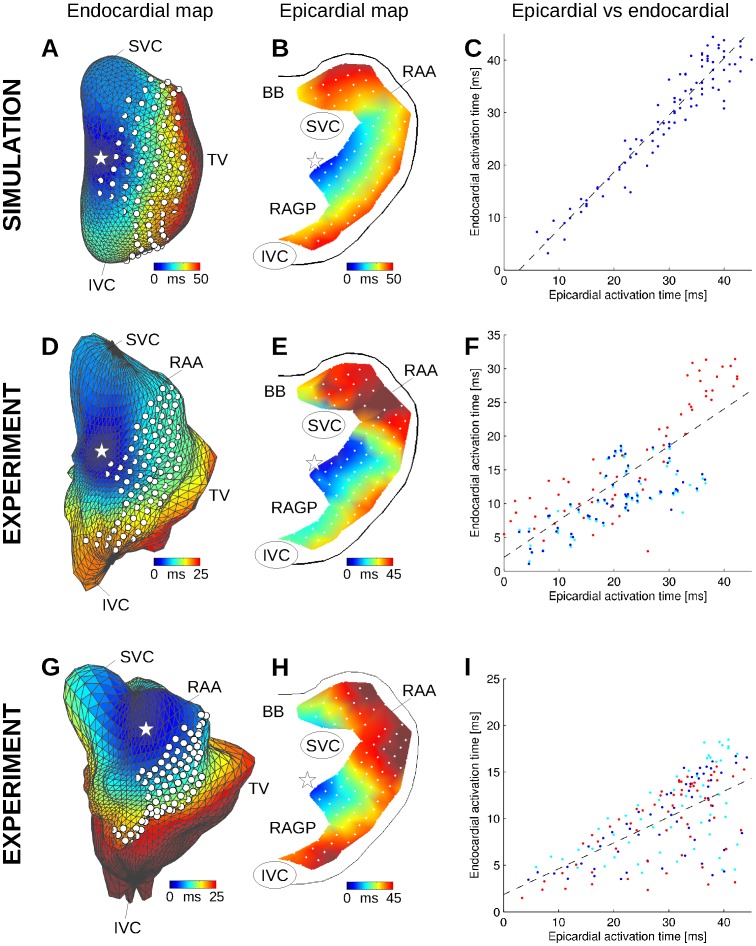
Endo- and epicardial activation maps in the computer model (A–C) and in the experiments (D–I). (A) Color-coded simulated endocardial activation map. White dots represent epicardial electrode positions. The white star denotes the earliest activation point. (B) Simulated epicardial activation map for the same atrial beat. (C) Epi- vs endocardial simulated activation times, along with the linear regression curve (dashed black line). (D) and (G) Experimental endocardial activation maps in two different dogs in control. (E) and (H) Corresponding experimental epicardial activation maps. (F) and (I) Epi- vs endocardial experimental activation times for the three beats (each shown with a different color) that served to identify epicardial plaque location. SVC: superior vena cava; IVC: inferior vena cava; RAA: right atrium appendage; BB: Bachmann’s bundle; RAGP: right atrium ganglionated plexus.

Figure 2 shows examples of simultaneous epicardial and endocardial mapping. The location of epicardial electrodes on the endocardial surface (white dots in Fig. 2D) were obtained by optimization of the correspondence between epi- and endocardial map (Fig. 2F). The overall epi- and endocardial activation patterns were consistent. The RMS difference in activation time was 10 ms and the correlation coefficient was 0.8 in the first dog (p<0.001; see Fig. 2F). Due to a less accurate geometrical reconstruction (Fig. 2G), the correlation coefficient was 0.63 (p<0.001) in the second dog, with an RMS difference of nearly 20 ms. The main inconsistency between epi- and endocardial mapping was found in the appendage, a region that is distant from the balloon catheter and at the same time poorly geometrically represented for the inverse problem. Note that the sinus beat originates from a focal point located outside the epicardial plaques.

In the computer model, exactly-known electrode location and geometry improved the consistency of the results. The endocardial activation map (Fig. 2A) was quantitatively similar to the epicardial map (Fig. 2B). Epi- and endocardial times (Fig. 2C) had a correlation coefficient of 0.96 and an RMS difference of 3.5 ms. The repolarization heterogeneities considered (changes in ACh concentration) had essentially no effect on the activation map at 100 bpm (maximum difference <0.8 ms).

With the exception of the appendage region, the computer model qualitatively reproduced experimental epi- and endocardial activation. Simulated and experimental epicardial maps (Figs. 2B vs 2E; 18 appendage data points excluded) had a correlation coefficient of 0.82 with an RMS difference of 6.8 ms (respectively 0.7 and 8.5 ms with all data points included). Since the computer model was derived from the experimental endocardial surface, endocardial activation could also be easily compared (data points in the valve and veins were excluded). Simulated and experimental endocardial activation times had a correlation coefficient of 0.91 with an RMS difference of 11 ms.

### Morphology of Bipolar Electrograms

**Figure 3:**
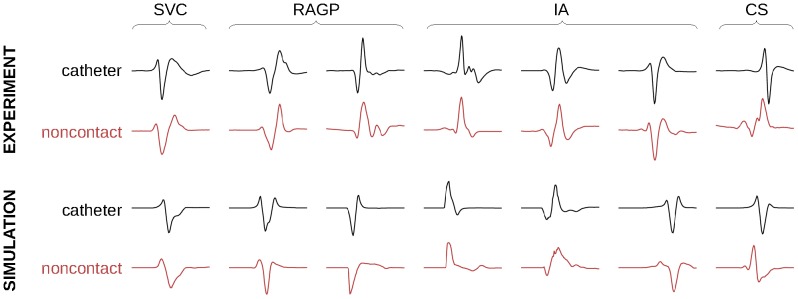
Morphology of direct-contact (catheter) and non-contact bipolar electrograms for 7 recording sites in the experiment and in the computer model (normalized signals). SVC: superior vena cava; RAGP: right atrium ganglionated plexus; IA: inter-atrial bundles; CS: coronary sinus.

Direct-contact bipolar electrograms (Fig. 3, first row) were recorded at the 7 endocardial sites shown in Fig. 1A. Since noncontact endocardial electrograms were unipolar, noncontact bipolar electrograms were reconstructed by computing the difference between noncontact unipolar electrograms measured at two locations in the vicinity (<5 mm) of the bipolar recording site (Fig. 3, second row). Because bipolar waveform depends on the unknown orientation of the bipolar electrode, the location of these two sites were adjusted to better match direct-contact recording. The correlation coefficient between contact and noncontact waveforms was 0.88±0.06 (range: 0.8 to 0.95), except near the coronary sinus where the value was lower.

In the computer model, bipolar electrograms were also computed as the difference between unipolar electrograms at two close sites. Dipole orientation was selected to generate the same types of waveform morphology observed in the experiment (Fig. 3, third row). Noncontact bipolar electrograms (Fig. 3, fourth row) were computed as the difference between two noncontact unipolar electrograms measured at exactly the same location as the direct-contact unipolar electrograms. Correlation coefficient between contact and noncontact waveforms was 0.85±0.10 (range: 0.69 to 0.97), waveform in the coronary sinus excluded.

### Area under the Atrial T Wave

**Figure 4:**
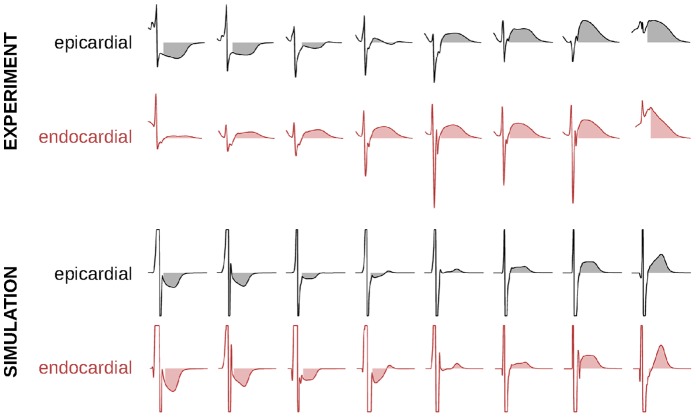
Examples of unipolar epicardial and noncontact endocardial electrograms measured (normalized) at corresponding epi- and endocardial sites (both experimental and simulated). The area under the atrial T wave is displayed as a shaded area. Simulated signals are saturated to highlight their atrial T wave.

**Figure 5:**
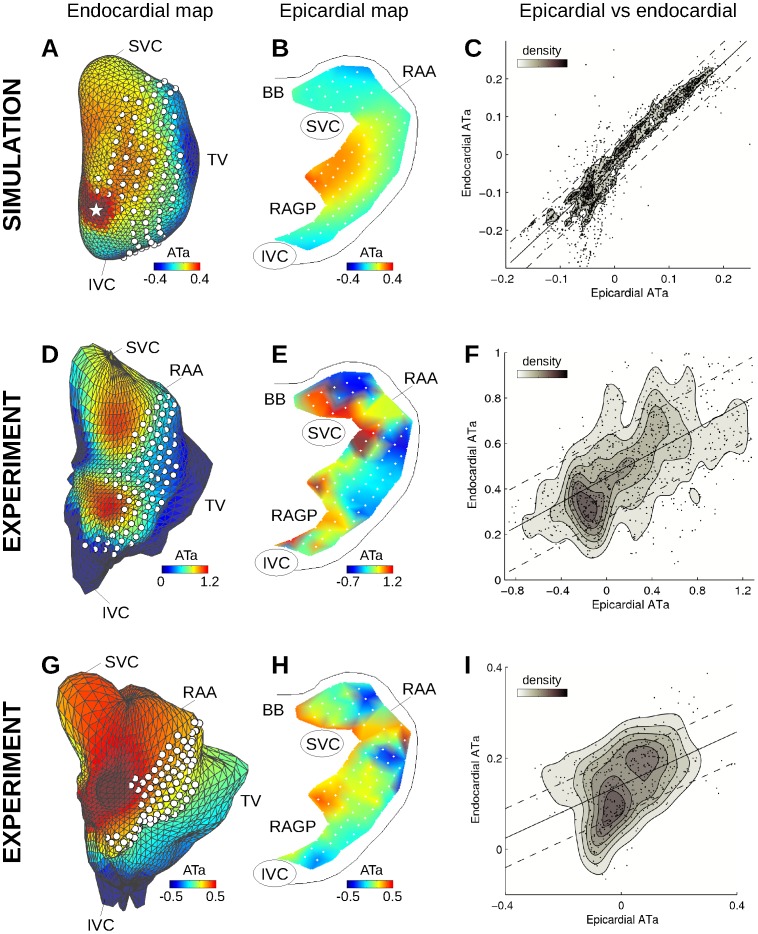
Endo- and epicardial ATa maps in the computer model (A–C) and in the experiments (D–I). (A) Color-coded simulated endocardial ATa map in the presence of repolarization heterogeneity with a radius of 3 mm around the white star. White dots represent epicardial electrode positions. (B) Simulated epicardial ATa map for the same beat. (C) Epi- vs endocardial simulated ATa for all simulations with different repolarization heterogeneity distributions, along with the linear regression curve and 50% confidence interval. Data point density estimated by kernel-based method is displayed as contour lines. (D) and (G) Examples of experimental endocardial ATa maps in two different dogs. (E) and (H) Experimental epicardial ATa map for the same beat. (F) and (I) Epi- vs endocardial ATa for all beats combined in each of the two dogs. SVC: superior vena cava; IVC: inferior vena cava; RAA: right atrium appendage; BB: Bachmann’s bundle; RAGP: right atrium ganglionated plexus.

The ATa provides a quantitative measure to assess atrial repolarization in experimental electrical recordings. Figure 4 illustrates the range of atrial T wave morphologies observed in the experiments and in the simulations at corresponding epi- and endocardial sites. Simulated atrial T waves had significantly lower normalized amplitude than the experimental ones (similarly to Vigmond et al. [12]) as revealed by smaller ATa values (Fig. 5), suggesting that canine atria contained stronger intrinsic heterogeneities than the model. Despite some differences in epicardial and noncontact endocardial atrial T wave morphology (notably atrial T wave did not become negative in experimental noncontact signals), epi- and endocardial ATa measurements appears to be correlated in these examples. Differences in signal amplitude, notably during the depolarization phase, may be due to inaccuracies in epi-endo electrode correspondence, or to volume conduction effects (e.g. Brody effect due to the higher blood conductivity [31]).

To further investigate this question, epi- and endocardial ATa maps were compared (Figs. 5D and E). The epi- and endocardial ATa patterns were found to be qualitatively comparable, except in the right appendage where signals were less reliable. The correspondence between epi- and endocardial ATa is summarized in Fig. 5F for all electrodes and 12 beats. To facilitate the interpretation, data point density was estimated using a kernel-based method [42] (kde2d Matlab script implementation by Z. I. Botev, available on Matlab Central website) and displayed as contour lines. The correlation coefficient between epi- and endocardial ATa values was 0.57 in the first dog (Fig. 5F) and 0.46 in the second (Fig. 5I). In both dogs, the hypothesis of no correlation between epi- and endocardial ATa was rejected at p<0.001.

The same analysis was performed on simulated data. Note that there was no attempt to match experimental repolarization properties. Instead, heterogeneities were introduced at predefined locations to assess whether these changes could be identified using mapping systems. Figures 5A and B show endo- and epicardial ATa maps for a simulated beat with a heterogeneity region of radius 3 mm around the white star in Fig. 5C. This region of increased repolarization gradients was characterized by higher ATa values. Epi- and endocardial ATa maps were consistent. The correlation coefficient between epi- and endocardial ATa values from the 22 simulations with different repolarization heterogeneity distributions was 0.92, as illustrated in Fig. 5C.

### Temporal Changes in Area under the Atrial T Wave

**Figure 6:**
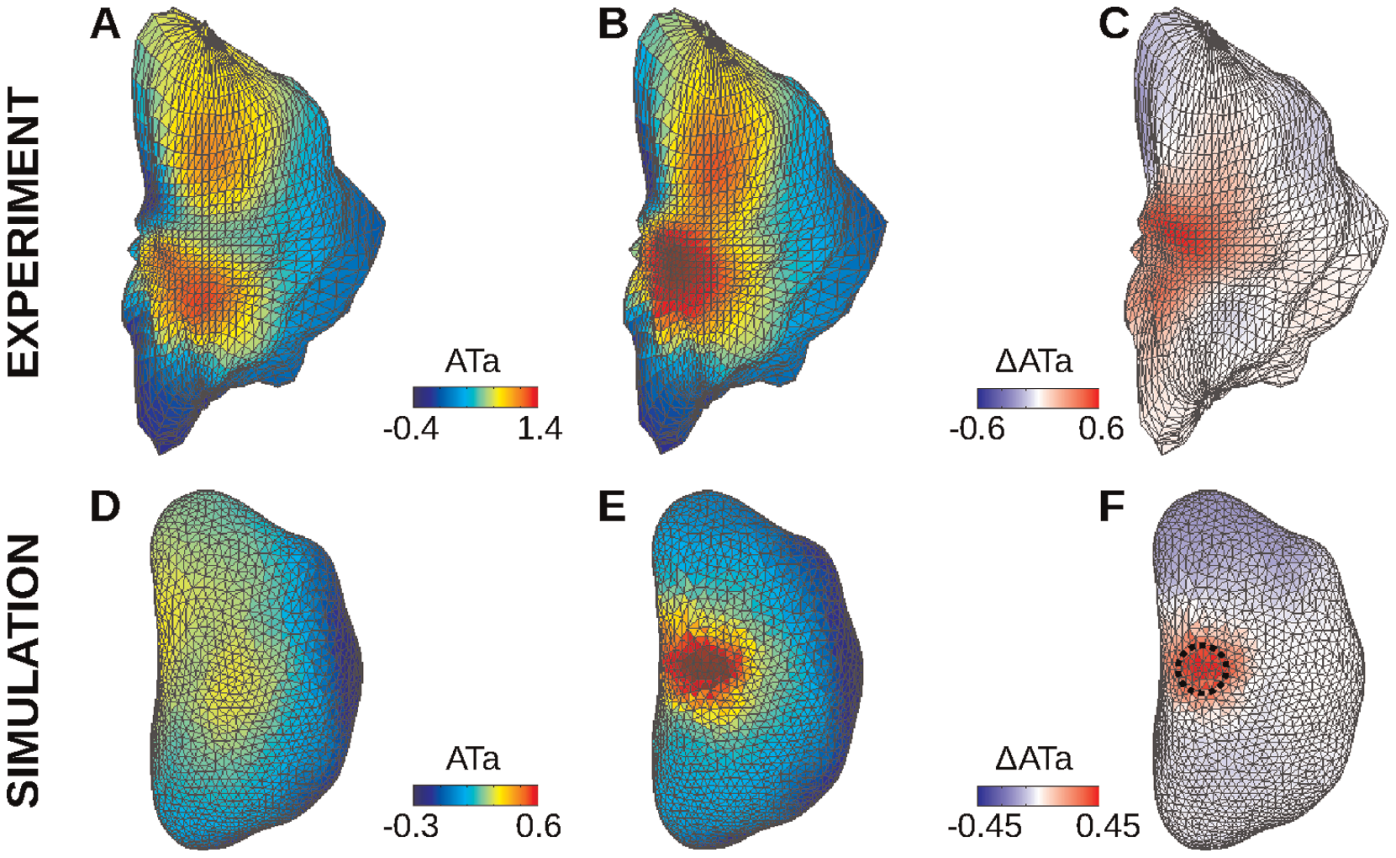
Endocardial ATa maps. (A) First experimental beat. (B) Another beat at a later time. (C) Difference between maps A and B. (D) Simulated ATa map during sinus rhythm in a uniform substrate. (E) Simulated ATa map with repolarization heterogeneity (3 mm radius in the right atrium ganglionated plexus; shown as a dashed circle). (F) Difference between maps D and E.

Experimental endocardial ATa maps were similar in the 12 analyzed beats. The correlation coefficient between any pair of them was always >0.9. The differences between ATa maps (separated by a few seconds or minutes) may reflect autonomic neural modulation [10]. To illustrate how endocardial ATa maps may be used to identify changes that occur outside the region covered by epicardial mapping, Figs. 6A–C displays ATa maps for two beats as well as their difference (ΔATa). On the ΔATa map, regions where changes occur can be easily identified.

In the simulations (Figs. 6D–F), repolarization gradients were induced by increasing ACh concentration in a circular zone, which affected ATa values. In the control beat (Fig. 6D), spatial variations in ATa were observed due to small repolarization gradients created by wavefront curvature or collision (this effect was |ΔATa| <0.04 in epicardial signals), and also due to distortions caused by the inverse problem. After subtraction, though, the pattern became clearer (Fig. 6F), thus enabling localization of the altered region. The distance between the center of the altered region and the maximum of the ΔATa map was <5 mm for the 4 regions in the right atrium ganglionated plexus and the inter-atrial bundles (Fig. 1B), and <9 mm for the 3 regions closest to the venae cavae. For the region near the superior vena cava, changes in repolarization were detectable only for a radius >3 mm. Otherwise, the radius of the region did not significantly affect the results in the range of parameters considered.

## Discussion

This paper presents a computational framework to evaluate the potential of noncontact mapping as a complement to epicardial mapping for studying neurogenically-induced changes in activation pattern and repolarization gradients. The simulation results can be seen as a best-case scenario as compared to animal experiment. The heart was not mechanically beating and the electrodes stay at fixed, exactly known locations. Note that in the experiment the EnSite system also makes that assumption. Neither conduction abnormalities [43] nor epicardium-endocardium dissociation [44], [45] were present in the model, which corresponds to the conditions we want to reproduce (neurogenic atrial fibrillation in a structurally normal heart). In this situation, noncontact mapping was able to correctly represent activation maps (depolarization, Fig. 2) and provide information about ATa maps (repolarization, Fig. 4), making it possible to identify and localize changes in repolarization properties (Fig. 6). In the control case with uniform membrane properties, ATa values derived from noncontact mapping were overestimated in the region were the balloon catheter was closest (Fig. 6D, where tissue properties are uniform), suggesting that the inverse problem introduces distortions that are noticeable in the repolarization phase. These distortions were however considerably reduced on ΔATa maps (Fig. 6). Note that the numerical methods and the inverse problem procedure could be improved [26]. Our intent was not to overperform the commercial package EnSite, but rather to illustrate in a computer model the possible distortions that the inverse problem can create. Further development of the model including more detailed anatomical representation of the trabecular structure and intrinsic variations in membrane properties would influence the baseline ATa map and require the use of ΔATa maps to properly interpret the data.

To compare epi- and endocardial data in the experiment, a major obstacle was to associate each epicardial electrode to an endocardial channel. Here, an optimization scheme was used to position the grid on the atrial surface. More reliable information about this correspondence may further improve the correlation between epi- and endocardial data (Figs. 2 and 4). Because of heart motion and limited details in geometrical reconstruction (especially veins, valve and appendage), this correspondence may however not be perfectly accurate. In the second dog with less accurate geometrical reconstruction, the correlation coefficient between epi- and endocardial data were indeed slightly weaker. On the other hand, when signals from the endocardial catheter and from noncontact endocardial channels at the same known location were compared, waveform morphology was relatively similar (Fig. 3), as reported in previous clinical and modeling works [18], [21], [26].

The relevance of epicardial ATa measurements for the identification of regions responding to autonomic neural modulation has been demonstrated in canine experiments and in computer models. Occasionally, epicardial plaques failed to identify the origin of neurogenically-induced ectopic beat when it occurred outside the region covered by the plaques. The extension to noncontact endocardial ATa would allow extracting information about regions that cannot be mapped on the epicardium. Results showed consistent ATa patterns in epi- and endocardial electrograms despite several limitations (Fig. 4). Noncontact endocardial signals, reconstructed from 64 electrodes, did not reproduce the full spectrum of atrial T wave morphologies observed in epicardial signals. For example, the ATa was always positive in noncontact signals, while many epicardial channels featured negative ATa values. Endocardial ATa maps were smoother, which facilitated the identification of regions with increased ATa (as in Fig. 6) but did not reproduce the large differences in ATa sometimes observed between neighboring epicardial electrodes. Epi- and endocardial ATa values were (moderately) correlated in the experiment, showing nevertheless that noncontact signals contain information about atrial repolarization. The accuracy of the prediction of epicardial ATa based on noncontact recordings seems limited. The ultimate goal is however to detect temporal changes in ATa (and thus repolarization) rather than obtaining an estimate of ATa values.

Although used here as reference, epicardial mapping has also its limitations regarding ATa measurement, notably in the appendage where the tissue had to be “flattened” to suture the plaques. Discrepancies between epi- and endocardial ATa could be caused by distortions in epicardial signals. In addition, there may be actual differences in epi- vs endocardial electrical activity due to complex (micro-) structures within the atrial wall. Finally, volume conduction in the surrounding medium is different from the epi- and endocardial side. This may affect the morphology of the atrial T wave. This confounding effect was avoided in the model, resulting in higher epi-endo correlation of ATa.

The application of noncontact mapping to animal experiments has been rare so far. The system has been designed for clinical setting. As a result, the atrial chambers need to be large enough to insert the balloon catheter (the balloon may not be maximally expanded if necessary), as is the case with large dogs. A more severe limitation (as in most unipolar recordings) is the contamination by ventricular activity. Atrio-ventricular node suppression and beat selection enabled us to extract atrial beats free of ventricular activity. For the application targeted in this paper (tracking the response to nerve stimulation in structurally normal canine atria in sinus rhythm before the onset of atrial arrhythmia), this limitation is not too restrictive. In order to monitor ATa on a beat to beat basis, efficient QRST cancellation algorithms dedicated to atrial electrograms need to be developed. This would be a prerequisite for clinical applications, which are beyond the scope of this paper. The validity of an extension to more irregular rhythms such as atrial fibrillation, particularly in the presence of uncoupling and fibrosis, is questionable due to the limited number of electrodes. The use of noncontact mapping system appears to be more appropriate for sinus rhythm or simple rhythms (e.g. flutter).

This issue of experimental reproducibility was only addressed by analyzing two dogs in this feasibility study. Although the results were consistent in the two dogs, more data will be needed to fully explore and validate the potential of the noncontact approach. In particular, the regions of higher ATa (as identified by noncontact mapping) were typically outside the zone covered by epicardial mapping (Figs. 5D and 5G), in agreement with the precise reason why noncontact mapping was undertaken (reentrant waves originated from outside the plaques [10]). This weakened the epi-endo comparison since the locations of maximal ATa values could not be compared.

Overall, noncontact mapping provides a complementary tool to achieve a more complete description of atrial electrical activity in canine experiments. Despite distance (balloon-atrial wall) and dimension reduction (64 electrodes), some information about atrial repolarization remains present in noncontact signals as supported by theoretical arguments relying on a computer model and some experimental evidences in a limited dataset comprising two dogs with structurally normal hearts. Further validation in the context of nerve stimulation (neurogenically-induced changes in repolarization) would reveal the ability of this technique to examine the activity preceding the onset of atrial arrhythmias in canine experiments.
